# The effectiveness of scoliosis screening programs: methods for systematic review and expert panel recommendations formulation

**DOI:** 10.1186/1748-7161-8-12

**Published:** 2013-07-24

**Authors:** Marie Beauséjour, Lise Goulet, Stefan Parent, Debbie Ehrmann Feldman, Isabelle Turgeon, Marjolaine Roy-Beaudry, Jose Felix Sosa, Hubert Labelle

**Affiliations:** 1 Sainte-Justine University Hospital Center, Unité de recherche clinique en orthopédie, 3175 Chemin Côte-Sainte-Catherine, Montréal, Québec H3T 1C5, Canada; 2 School of Public Health, Université de Montréal, 7101 Avenue du Parc, 3rd floor, Montréal, Québec H3N 1X9, Canada; 3 Department of surgery, Faculty of medicine, Université de Montréal, PO Box 6128, Succ. Centre-Ville, Montréal, Québec H3C 3J7, Canada

**Keywords:** Scoliosis, Mass screening, Adolescent, Program evaluation, Systematic review

## Abstract

**Background:**

Literature on scoliosis screening is vast, however because of the observational nature of available data and methodological flaws, data interpretation is often complex, leading to incomplete and sometimes, somewhat misleading conclusions. The need to propose a set of methods for critical appraisal of the literature about scoliosis screening, a comprehensive summary and rating of the available evidence appeared essential.

**Methods:**

To address these gaps, the study aims were: i) To propose a framework for the assessment of published studies on scoliosis screening effectiveness; ii) To suggest specific questions to be answered on screening effectiveness instead of trying to reach a global position for or against the programs; iii) To contextualize the knowledge through expert panel consultation and meaningful recommendations. The general methodological approach proceeds through the following steps: Elaboration of the conceptual framework; Formulation of the review questions; Identification of the criteria for the review; Selection of the studies; Critical assessment of the studies; Results synthesis; Formulation and grading of recommendations in response to the questions. This plan follows at best GRADE Group (Grades of Recommendation, Assessment, Development and Evaluation) requirements for systematic reviews, assessing quality of evidence and grading the strength of recommendations.

**Conclusions:**

In this article, the methods developed in support of this work are presented since they may be of some interest for similar reviews in scoliosis and orthopaedic fields.

## Background

The value of scoliosis screening programs was often debated and is still a controversial issue as indicated by the SOSORT 2007 positional statement, Plaszewski’s 2012 review and 2012 historical article by Linker [1-3]. In most cases, scoliosis screening was achieved through mass systematic examination of children, in the school environment, searching for back asymmetries. These programs are still in operation in some countries but were discontinued in many others since the 1980s. Indeed, the British, American and Canadian Preventive task forces, who based their decisions on the best available evidence at the time, did not recommend the use of these programs. The two main issues raised by the Canadian task force were the ability of the detection procedure to detect the condition and the ability of the available treatment intervention to achieve a favourable outcome [4,5].

In 1993, the United States Preventive Services Task Force concluded that there was insufficient evidence to recommend for, or against, screening but, in 2004, it recommended against the routine screening for AIS, and maintained this position [6] despite its continuing support from medical bodies (American Academy of Orthopedic Surgeons, Scoliosis Research Society, Pediatric Orthopedic Society of North America and American Academy of Pediatrics) and other interest groups [7]. The Canadian position (2003) is that insufficient evidence exists for a recommendation to be made [8].

Literature on scoliosis screening is vast. There are a few reviews and expert opinion papers, guiding the reflection on the clinical benefits of such initiatives and also, possible burden and societal costs of the programs [9-12] However, published studies consist of mainly prevalence studies or reports on screening programs set-up and process with very few controlled trials and comparative studies.

A meta-analysis published by Fong et al. [13] has shed light on the clinical effectiveness of scoliosis screening programs. This rigorous review focused on scoliosis prevalence, referral rates and positive predictive values of the tests as main outcomes, from 36 published retrospective cohort studies. However, other dimensions of effectiveness should also be considered to get a more complete assessment. Sabirin et al. [14] conducted a systematic review that was an interesting attempt to widen the scope of previous reviews by exploring four themes: effectiveness of scoliosis detection, consequences on surgical treatment, cost/cost-effectiveness and diagnostic accuracy. However, the review could have been more extensive in terms of included studies and assessment of the study quality.

Because of the paucity of controlled trials in this field, the observational nature of available data and methodological flaws, data interpretation is often complex, with the inherent risk of leading to incomplete and even misleading conclusions.

The need to propose a set of methods for critical appraisal of the literature about scoliosis screening, a comprehensive summary and rating of the available evidence appeared essential. More specifically, our objectives were:

i) To propose a framework for the assessment of published studies on scoliosis screening effectiveness;

ii) To suggest specific questions to be answered on screening effectiveness instead of trying to reach a global position for or against the programs;

iii) To contextualize the knowledge through expert panel consultation and meaningful recommendations.

## Methods

**Figure 1:**
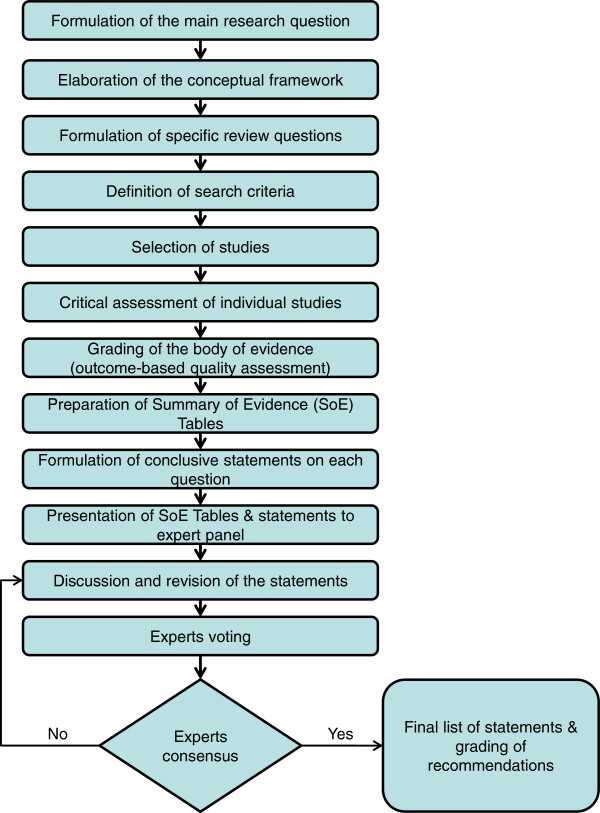
Flowchart of the general methodological approach.

The general methodological approach proceeded through the following steps (Figure 1):

a) Elaboration of the conceptual framework;

b) Formulation of the review questions;

c) Identification of the criteria for the review;

d) Selection of the studies;

e) Critical assessment of the studies;

f) Results synthesis;

g) Formulation and grading of recommendations in response to the questions.

This plan follows the GRADE Group (Grades of Recommendation, Assessment, Development and Evaluation) requirements for systematic reviews, assessing quality of evidence and grading of the strength of recommendations [15-17]. Some modifications to the tools were proposed because of the specific study context and available material but also in consideration of our capabilities as a research team and preferences of the involved experts. The intention was not to modify the GRADE approach (adopted by the Cochrane Collaboration [18]), that has proven its worth and usefulness, but, to describe, in our desire to stay as close to it as possible, the encountered difficulties and alternative ways to proceed. The review protocol was developed in such a way to enable the authors to follow the PRISMA statement when reporting the review methodologies and results [19].

### a) The conceptual framework

The main research question of the review was: What is the evidence in the literature on the effectiveness of scoliosis screening programs in the adolescent population?

**Figure 2:**
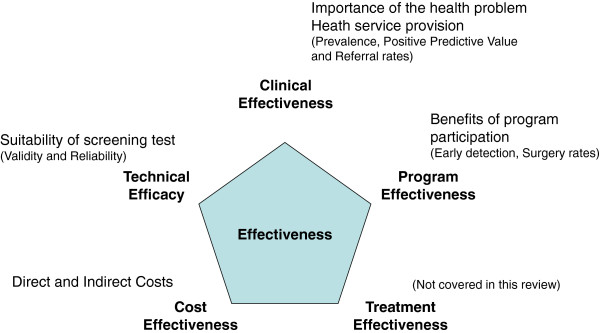
Conceptual framework of Effectiveness.

To conceptually define Effectiveness, we relied on Last’s definitions of Efficacy and Effectiveness. According to Last, Effectiveness is “the extent to which a medical intervention does what it is supposed to do”. In contrast, Efficacy concerns “the extent to which an intervention produces benefits under ideal circumstances” [20,21]. We referred to the classical Wilson and Jungner criteria for the appraisal of screening programs [22] and the reviewed criteria by the UK National Screening Committee [23] to define five main dimensions of effectiveness: Technical Efficacy relates to the validity and reliability of the tests; Clinical Effectiveness describes the importance of the health problem and the consequences of screening on patient management and the health system; Treatment Effectiveness concerns the benefits for the patients of the available treatment modalities; Program Effectiveness refers to the benefits for the patients who adhere to the screening programs, and these benefits are balanced against costs for the society in Cost-Effectiveness. These dimensions are evaluated under regular practice circumstances, except for Technical Efficacy which may be evaluated under experimental protocols on selected samples of participants. Our conceptual framework of Effectiveness is depicted in Figure 2.

This conceptual model was *a posteriori* empirically supported by the findings of what was actually available in the literature in this field.

It is important to mention that the assessment of Treatment Effectiveness, which would have been the 5th dimension of effectiveness to be considered based on the model, was not completed in this review. Even though the existence of an effective treatment is of capital importance in the decision to screen or not for the disease of interest, the authors considered that this topic would necessitate a systematic review on its own. Despite the need for such an evaluation [24], in the specific context of this review, it was decided to focus solely on the screening process.

### b) Research questions

Seven specific questions were addressed, in relation to the main dimensions of effectiveness that were covered. The following questions were originally derived by the authors and were elaborated in correspondence to the criteria for program appraisal of the UK National Screening Committee [22,23] comprising characteristics of the condition, of the screening tests and of the program.

Technical Efficacy:

▪ What is the best technique/tool for scoliosis screening in terms of validity and reliability?

Clinical Effectiveness:

▪ Is Adolescent Idiopathic Scoliosis a prevalent disease?

▪ What is the proportion of patients referred to orthopaedics with suspected AIS from screening programs?

▪ What is the probability of actually having a diagnosed scoliosis if tested positive on screening?

Program Effectiveness:

▪ Are screen detected scoliosis patients younger and less severely affected at time of detection and diagnosis than otherwise detected patients?

▪ Are screen detected scoliosis patients less likely to be recommended for surgery than otherwise detected patients?

Cost-Effectiveness:

▪ What is the cost of screening for scoliosis and does it seem cost-effective/cost-beneficial?

### c) Criteria for considering studies for this review

#### Study design

All studies published from 1950 to mid-2010 related to screening programs for scoliosis were considered. This included all randomised controlled trials, controlled clinical trials, comparative studies of any kind, evaluation and validation studies, as well as all sorts of epidemiologic studies (including prevalence studies).

#### Study population

The main target population was composed of adolescents aged between 10 and 18 years old. Programs focusing only on infantile or juvenile scoliosis were excluded. In addition, some programs that were designed to screen younger population were included if they provided separate data for the adolescent group.

#### Interventions

The interventions under consideration were all programs designed to favour early detection of AIS by systematic examination, of presumed healthy adolescents in the school or community setting, searching for back asymmetries. The following tests were considered: visual inspection in upright and/or forward bending position with/without objective measurement of back asymmetries using the scoliometer, an inclinometer, a plane and a ruler, or by analysis of Moiré topography fringe images. The test(s) could be performed either by a nurse, a physiotherapist, a sport instructor, a school doctor, a general practitioner, a paediatrician or an orthopaedist (including residents in these medical fields).

#### Comparisons

When available, “non-screened” patients or otherwise detected patients were considered as control groups. Their cases may have been brought to the attention of the orthopaedist either by parent suspicion or detection by another health professional. They were incidental findings not recruited through a specific program targeting the early identification of scoliosis cases. They could also be historic controls before a program was established or geographic controls in regions where the program was not deployed.

#### Measures and outcomes

The measures used in this review were related to the dimensions of Effectiveness previously defined (Figure 2). Studies included under the topic Technical Efficacy focused on the value of the detection methods. Therefore, the measures of interest are intra and inter-observer reliability (assessed by the computation of intraclass correlation or kappa coefficients) as well as validity of the detection methods or tests which may be operationalized as sensitivity, specificity, positive and negative predictive values in test samples. Clinical Effectiveness, is the most commonly studied dimension in the scoliosis screening literature. In concordance with Fong et al. 2010 [13], we considered three main measures: scoliosis prevalence, referral rate and positive predictive value as consequences of screening programs (in real world settings). Studies classified under the topic Program Effectiveness were those that focused on patient-oriented outcomes or the program benefits for the patients’ population in comparison to non participants. The two main outcomes were patient characteristics (maturity and curve severity) at time of detection and diagnosis, and reduction in the number of surgeries for scoliosis. Program costs reports (including detailed cost-effectiveness analysis if any) included: costs per child screened, per patient diagnosed and per patient treated and eventually cost-benefits/cost-effectiveness ratios. The considered costs were direct and/or indirect costs of programs, for screening only and/or subsequent management.

### d) Search methods and selection of studies

A literature search using both MeSH words and free-text keywords was independently done by two content knowledgeable research assistants and validated by a librarian at Université de Montréal. Four databases were searched: Medline (1950 to July 2010), Embase (1980 to July 2010), CINAHL (1980 to July 2010) and the Cochrane Central Registry of Controlled Trials (EBM Reviews, Wolter Kluvers up to 2nd quarter 2010). In the searches, the subject has been described in accordance with the PICO method [25] using 4 concepts (the comparisons were not included in the search strategies) linked together using logical operator “and”: the disease (Scoliosis, Spinal deformities, Back asymmetry, etc.), the population (Child, Adolescent), the intervention (Screening program, Bending test, Moiré topography, etc.) and the outcome (Prevalence, incidence, epidemiology, etc.), in all contexts (not restricted to school screening). In the queries, the synonyms or different terms describing the same concept or different spelling of these words were linked together using logical operator “or”. Detailed labelling of a query example is presented in Table 1. No *a priori* language limits were used in the search queries. The bibliographies of all selected articles and task forces reports were searched for additional relevant references. References were imported into the software program database EndNote X3.

**Table 1:** Example of search queries, Medline 1950 to July 2010 (Lunched on July 23rd 2010)

**Searches**	**Results**
1. Scoliosis/	11886
2. Spinal Curvatures/	403
3. screen*.mp.	362323
4. depistage.mp.	3288
5. Mass Screening/	68020
6. Program Evaluation/	35624
7. Child/	1177573
8. Adolescent/	1361381
9. (prevalence* or incidence* or epidemiolog* or detection* or adams or moire or scoliometer*).ab,ti.	1163122
10. (back adj5 (asym?etry or asym?etrical)).ab,ti.	46
11. (forward adj5 (bend or bends or bending)).ab,ti.	361
12. scolio*.mp.	14190
13. 1 or 2 or 10 or 12	14495
14. 3 or 4 or 5 or 6 or 9 or 11	1471753
15. 7 or 8	1911903
16. 13 and 14 and 15	1247
17. 13 and 14	1714
18. limit 17 to "all child (0 to 18 years)"	1291
19. 16 or 18	1291

Note: *=truncation symbol, ?=spelling variants, ab=abstract, ti=title, mp=abstract, title, author keywords and keywords plus, adj5=words in the same sentence.

Two review authors evaluated the search results by reading titles and abstracts of all non redundant articles found. Papers that concern detection of non idiopathic scoliosis in related syndromes, genetic screening of scoliosis, or scoliosis surgical treatment, were discarded. From this first selection, potentially relevant articles were retrieved in full text for complete reading and eventually retained for further analysis. Although, the search strategy included all languages, only studies reported in English or French were considered for full text review (review of publications of interest in other languages were limited to the abstract, which is a limitation of the study in terms of international coverage). In case of disagreements in the inclusion of a study, a third review author was contacted to resolve the conflict.

**Figure 3:**
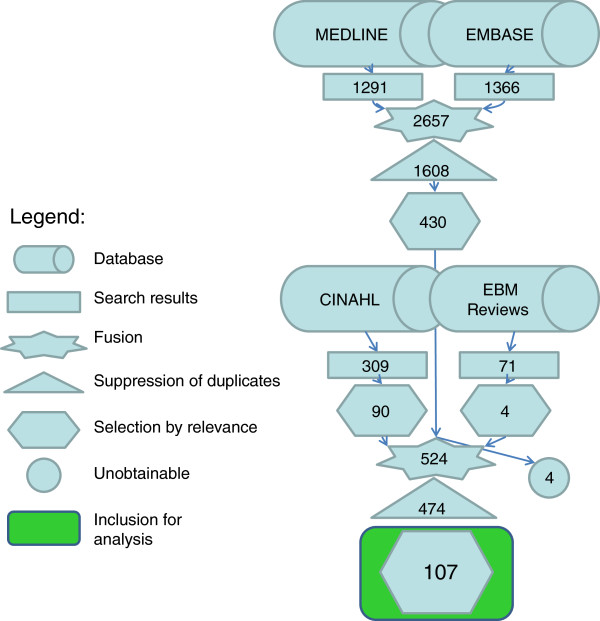
Study selection algorithm.

The detailed algorithm for selection of studies is depicted in Figure 3. From the extensive search on the four databases with a validated query, a total of 107 non redundant relevant papers were identified and thoroughly analysed for the first four dimensions of effectiveness.

### e) Critical appraisal of the studies

All selected studies were distributed for review to three reviewer teams, each consisting of two review authors with different backgrounds, an orthopaedist and an epidemiologist. A standardized data extraction form was prepared from Excel spreadsheets. Its content was thoroughly discussed for interpretation issues and pre-tested on three representative studies with all six review authors. Key findings were summarized in narrative format for each article and included: the study design, the sample size, the details of the specific intervention (tools, personnel, setting, repetitions,…), description of the main outcomes, measures of intervention effects (outcomes), the key message and the main conclusions by the original authors, the paper’s strengths and flaws, assessment of risk of bias as well as critical appraisal of the conclusions and clinical significance according to the review authors.

#### Assessment of methodological quality

Strength of evidence was assessed in two manners. First, from each study, the more recent version of Downs and Black tool [26] was used. It is a checklist for the assessment of the methodological quality of randomised and non-randomised studies of health care interventions with known satisfactory test-retest and inter-rater reliability [26]. Study assessment covers 4 topics: quality of reporting (10 items), internal validity (13 items), external validity (3 items) and power (2 items). This tool has been previously used by our team in the past [27-29]. Review authors clarified any item interpretation issues and inter-rater agreement was satisfactorily tested on three articles prior to this study.

As Sabirin et al. [14] suggested, effectiveness and diagnostic studies may have different requirements for assessment. Therefore two questions from the original Downs and Black scale were modified. Items related to the measure and adjustment for confounders and blinding of the intervention were replaced by review authors formulated questions about quality assessment of the reliability and validity experimental protocols regarding expertise of the screeners and standardization of the screening processes.

For each paper total score was obtained by consensus of two review authors. To give a more qualitative interpretation to the Downs and Black scores, we have proposed categorization of studies as *high, moderate, low or very low quality* according to their scores. This was previously suggested by others [30-32] who used quartiles distribution or equal-size intervals to interpret the scores. Here it was decided that the paper should at least clearly describe the characteristics of patients, the intervention and the main outcomes, use a representative intervention in a representative population, with accurate measures and appropriate statistical tests, with some consideration of confounding, and sufficient power. Moreover, using randomization, blindness, comparison groups recruited from the same population over the same period, having presented the distributions and proper adjustment for confounders, and having explicitly reported a power calculation, would make the study high quality (maximum score on items 5, 14, 15, 22–25, 27). Under these criteria, the proposed categorization was: more than 20 = high quality, 15–20 = moderate, 6–14 = low and less than 6 = very low.

Secondly, we assessed overall quality of the evidence of the body of knowledge related to each measure/outcome as recommended by the GRADE system. According to GRADE, evaluating quality of evidence for health care question is to determine the extent to which we are confident that an estimate of effect is correct for a particular outcome [17,33]. GRADE defines eight criteria for quality of evidence assessment. For greater applicability in our review context, we have grouped the criteria under three main categories and used Ebell’s [34] approach to upgrade the level of evidence of the corpus based on “quality criteria”: strength of association, dose–response gradient, effect of bias and possible confounders, “quantity criteria”: number of studies, size of population, directness of evidence and precision, and finally “consistency criteria” or homogeneity of results. The strength of evidence of the body of knowledge in this review was considered to start as low (observational studies) and was upgraded or downgraded according to our interpretation of Ebell’s criteria.

### f) Summary of findings

The GRADE group has proposed a general template for summary of findings (SoF) which is a convenient way of combining both main results and quality of evidence assessment in a table, to increase usability of the review and support decision making. Summary of findings tables were compiled under each effectiveness dimension that was evaluated, for each considered measure/outcome, and for each comparison in intervention modalities available in the studies.

Based on their review, the authors proposed “recommendations” or more appropriately conclusive statements derived from the literature which could impact practice and also future research.

To provide clinical meaning to the conclusive statements, we recruited orthopaedic surgeons in active practice with the paediatric population to participate in an expert panel modified Delphi session. Panel members were presented with the conceptual framework, the methods, the SoF tables and the proposed statements for each topic. SoF tables were clearly presented in an interactive manner using Power Point software. Upon request, detailed individual study evaluation and full text articles were provided prior to the panel session. Documentation was provided by e-mail and through a dedicated Wikispace.

Panel members were invited to ask questions about the relevant evidence and openly discuss the content of each statement (one at a time). A moderator (one of the review authors) stimulated participation of members into the debate. Modifications were considered and integrated in real-time. Discussions continued until final agreement on the statements’ wording and content.

### g) Grading the recommendations

According to the GRADE system, recommendations may be considered as strong or weak and this decision emerges from the consensus of an expert panel about the balance between benefits and downsides of the adherence to the proposed statements [16,17].

Upon agreement on a final version of the statements, panel members were invited to vote on the strength of the recommendations. Polls were conducted privately and anonymously, either on paper (first panel) and using an audience response system (Turning Point Technologies – second panel). Two expert panels accepted to participate in this study. The first one was composed of 11 orthopaedic surgeons of the *Quebec Scoliosis Society* brought together for a workshop session of the Annual meeting of the Society on October 2010 in Burlington, Vermont. The second panel was composed of 10 orthopaedic surgeons, members of the *Canadian Paediatric Spinal Deformities Study Group,* attending their bi-annual meeting in Quebec City in March 2011.

## Discussion

In this work, there was concern to clearly define the research questions and especially the dimensions of the Effectiveness concept in order to adequately cover the subject and try to go further and above the work done by the task forces in the 1980s. Moreover, we aimed at providing answers to specific questions on elements of programs (screening modalities, effects on surgery rates,…) instead of a global statement for or against systematic screening. This approach is more likely to support serious reflection on secondary/tertiary prevention of scoliosis and elements that may be integrated into prevention programs and primary care management of AIS patients.

A clear definition of the concepts under evaluation, and relying on a conceptual model of effectiveness appeared both sound on a theoretical basis and very helpful to support search strategy, studies selection and computation of evidence.

We used a standardized tool, the Downs and Black checklist to grade the strength of evidence, which was recommended in West et al. and Deeks et al. reviews [35,36]. It was here adapted on two items for the assessment of screening tools. Although the Downs & Black tool is time-consuming to apply and requires considerable epidemiology expertise, it has not been found difficult to apply in this review. Unfortunately, the modified Cochrane Collaboration Risk of Bias tool for use in nonrandomized studies [37] was not ready to use when we started this work. Review authors were trained in the Downs and Black tool prior to data analysis. Working in mixed-teams of orthopaedic surgeons and epidemiologists was of great benefit to put together complementary strengths and expertise, even though it needed some time investment in the beginning to “speak the same language”, especially on a subject which traditionally lead these professionals to opposite views [38].

The body of knowledge in this field is composed of observational studies only - a design that may suffer from many biases. Reported conclusions by the authors were then considered with caution.

The available data were not seen as appropriate for computation of pooled estimates. Therefore no statistical analysis was performed; neither did we conduct sensitivity or heterogeneity analysis of data. The production of a qualitative review is of course limited, but it was considered an appropriate way to proceed considering the observational nature of the available literature in the field.

Access to recognized effective treatment is a major concern in any decision about a screening program. We acknowledge the limitation of not having considered scoliosis treatment, and brace management, in particular. The literature on the effectiveness of brace treatment is vast and requires a systematic review of its own. As a very good starting point, one could consider the three published systematic reviews: Negrini et al. 2010 [24], Lenssinck et al. 2005 [39] and Rowe et al. 1999 [40]. Despite the publication of numerous case series and a certain number of comparative studies, best evidence on brace effectiveness is still pending. We believe that any serious effort to systematically summarize quality evidence on brace effectiveness should wait for the publication of the results from current NIH clinical trial on bracing, the BRAiST study [41]. In addition, trials on scoliosis-specific exercise regimes are also in progress (e.g. [42]).

Finally, this work resulted in the elaboration of recommendations, both for practice and for research. They are presented as a report of the current knowledge and a position statement on the effectiveness of screening programs. They should not be perceived as clinical practice guidelines *per se* or program orientations. We have compiled the evidence with great caution and we are confident that the conclusions correspond directly with the results of the literature analysis. We wanted the process to be based on exhaustive and rigorous appraisal of scientific knowledge but also to be contextualized through expert consultation. The strength of the evidence was clearly stated in the recommendations and therefore, the panel members considered the quality of the evidence when grading their confidence in the recommendations. There was no explicit evaluation of possible harms of the screening procedures and we relied on expert opinions for globally considering perceived harms. We believe that the submission of the recommendations to expert panels is a good way to ensure that recommendations have clinical meaning without being beyond the scope of the review.

## Conclusions

The literature on scoliosis screening is vast. The need to propose a set of methods for critical appraisal of the literature about scoliosis screening, a comprehensive summary and rating of the available evidence appeared essential. The proposed methods include several innovations: i) A clear definition of the concepts under evaluation by relying on a conceptual model of effectiveness for sound theoretical basis and support for search strategy, studies selection and computation of evidence. ii) The formulation of focussed questions on specific elements of programs (screening modalities, effects on surgery rates,…) that the review intend to answer, instead of trying to get a global statement for or against systematic screening as the only endpoint. An approach that we believe is more likely to support serious reflection on secondary/tertiary prevention of scoliosis and elements that may be integrated into prevention programs and primary care management of AIS patients. iii) The first use of the GRADE Group approach in literature assessment in the field of scoliosis screening. iv) The adaptation of the Downs & Black tool for the evaluation of effectiveness of diagnostic studies. v) The elaboration of “recommendations” or conclusive statements, both for practice and for research, derived from the literature review and contextualized by expert panels.

Methods developed in support of this work may be of interest for similar reviews in scoliosis and orthopaedic fields where the body of knowledge is mainly composed of observational studies.

## Competing interests

The authors of this article have no financial or personal relationship with other people or organizations that could inappropriately influence (bias) their work, except for Hubert Labelle and Stefan Parent who have the following financial relationship to disclose: Stock ownership with Spinologics Inc.

## Authors’ contributions

**MB** conceptualized and designed the study, performed the literature search and was involved in literature assessment. She drafted the initial manuscript and approved the final manuscript as submitted. **LG** is the thesis director of MB. She supervised study design, especially in the adaptation of the evaluation tools, and was involved in literature assessment. She revised the manuscript and approved the final manuscript as submitted. **SP** was involved to the choice and validation of the evaluation tools. He was also involved in literature assessment. He revised the manuscript and approved the final manuscript as submitted. **DEF** contributed to the definition of the conceptual framework and was involved in literature assessment. She approved the final manuscript as submitted. **IT** is a research associate with the team. She coordinated data collection. She also managed the collaboration with all the other participants (members of the *Quebec Scoliosis Society* and the *Canadian Paediatric Spinal Deformities Study Group*)*.* She approved the final manuscript as submitted. **MRB** is a research associate with the team. She contributed to the study design and literature search. She approved the final manuscript as submitted. **JFS** was involved in literature assessment and approved the final manuscript as submitted. Members of the *Quebec Scoliosis Society* and the *Canadian Paediatric Spinal Deformities Study Group.* As experts in scoliosis research and treatment, they were involved in the validation of the recommendation elaboration process and participated in the expert panels for the grading of recommendations. **HL** is the thesis co-director of MB. He supervised study design, contributed to the definition of the conceptual framework and was involved in literature assessment. He also critically revised the manuscript, and approved the final manuscript as submitted. All authors read and approved the final manuscript.
